# Long-Term Effectiveness of Parent Education Using the “Baby Oral Health” Model on the Improvement of Oral Health of Young Children

**DOI:** 10.1155/2013/137048

**Published:** 2013-11-10

**Authors:** Gajanan V. Kulkarni

**Affiliations:** Department of Paediatric & Preventive Dentistry, Faculty of Dentistry, University of Toronto, 124 Edward Street, Room 455D, Toronto, ON, Canada M5G 1G6

## Abstract

*Purpose.* To determine the long-term effectiveness of comprehensive education given to parents and caregivers with respect to the incidence of preventable oral diseases, utilization of dental services, and retention of knowledge related to oral health. *Methods.* Group presentations on oral health were conducted for caregivers of infants (*n* = 161) using an interactive audio-visual aid. Followup occurred at 18 months. A comparison group (*n* = 181) was enrolled from the same community groups. Chi-square and Fisher's exact tests were used to analyze findings. *Results.* There was a difference in caries incidence, knowledge levels of caregivers, and utilization of dental services (*P* < 0.05) when comparing the SGB to the SGFU. *Conclusions.* One-time exposure to parent education using a comprehensive interactive audio-visual aid has an effect on reducing caries incidence and increasing dental utilization. While most knowledge is retained by parents, there is some attrition in the information retained over an 18-month time period. This emphasizes the importance of repeated reinforcement of the same concepts over a shorter time span.

## 1. Introduction

Providing comprehensive education to caregivers for the promotion of good oral health in their children is now termed as anticipatory guidance. Anticipatory guidance, as defined by Nowak and Casamassimo [1], is the “process of providing information about children to their parents by alerting them to impending changes, teaching them their role in maximizing their children's developmental potential, and identifying their children's special needs.” Traditional preventive strategies have been implemented after deleterious habits have progressed, and these strategies have shown to be limited in their success rate over long periods of time [2, 3]. The timely manner in which this information is given to caregivers is a crucial point in this education strategy.

 Anticipatory guidance has been used in the medical community in its campaign to encourage each patient to have a medical home. A medical home is an approach to providing comprehensive primary health care that is easily accessible, culturally sensitive, and family centred in a compassionate manner [4]. Studies of the medical home have shown that having a regular source of medical care has decreased the utilization of hospital emergency facilities [5]. The literature has also shown that having a preventive dental visit by the age of one increases future preventive visits and decreases future restorative and emergency room visits [6]. 

 Traditional preventive strategies have shown an increase in knowledge and attitudes with dental education, but this has not translated into changed behaviour patterns in the long term [3]. In contrast there are other reports of successful health education programs. For example, a randomized controlled study of the effects of a pedagogical device targeted to prevent hypoglycemia proved to be a cost-effective educational tool [7]. The development of an anticipatory guidance model via a comprehensive audio-visual aid was achieved by Alsada et al. [8]. 

The aim of the study was to determine the long-term effectiveness of our anticipatory guidance model. Specifically, we determined and compared three outcomes: (1) the long-term retention of knowledge; (2) access of dental care by the caregivers for their children; (3) the incidence of preventable oral diseases such as dental caries.

## 2. Materials and Methods

The two-cohort study was approved by the research and ethics committees of the University of Toronto. The anticipatory guidance model used in the study was an interactive presentation that included use of a DVD termed “Baby Oral Health” in a community-oriented setting. This DVD was designed as a tool to provide comprehensive education regarding infant oral health in high-risk populations. Unlike existing education materials, this aid provides a comprehensive, self-directed, and evidence-based approach to the promotion of infant oral health. The topics of the video included the role of a healthy pregnancy, stages of tooth development, early childhood caries (baby bottle tooth decay), trauma, nutrition, oral hygiene, fluoride, oral bacteria, nightly feeding habits, oral habits, the first dental visit, and regular dental visits. This video was developed and previously tested in a pilot project for its effectiveness in infant oral health education [8]. The presentations were performed by one dentist at city-operated child care centres or Ontario Early Years Centres in Toronto. 

To assess infant oral health knowledge, each caregiver was asked to complete a questionnaire relating to the presentation. This questionnaire was edited from the questionnaire used in the pilot study [8]. There were two types of data collected to assess the effectiveness that the intervention had on preventable oral diseases. The first was the dental screening performed by one dentist in the knee-to-knee position. An overhead light and examination gloves were used. Caries was defined as visibly cavitated lesions. The second was the completion of an assessment form to review any high-risk behaviour. Topics covered in the assessment form included demographics, birth history, diet and nutrition, fluoride, oral habits, injury prevention/trauma, oral development, oral hygiene, and dental visits. In order to determine the model's effect on dental utilization, each caregiver was provided a referral form which listed paediatric dentists in their vicinity. 

Caregivers voluntarily participated in the study based on information provided by the directors of participating centers to various parent groups. Any and all parents with appropriate age children or expectant parents who consented to participation in the study were enrolled. There were no exclusion criteria as this program is intended for all parents with young children.

The schematic below outlines the study design; arrows denote the statistical comparisons made:

 
Study  group:  enrolled  atbaseline  (n=161;  SGB)⇔Study  group  followupat  18 mo  (SGFU)0000⇕00000Comparison  group⇔Enrolled  at  followup(CG)

For the study group at baseline (SBG; *n* = 161), the assessment forms were completed by the caregiver prior to the start of the presentation. The anticipatory guidance presentation was delivered. The caregivers completed the questionnaire immediately after the presentation, and then their children participated in the dental screening. The study group was followed up after an 18-month time period (SGFU; *n* = 161). The follow up consisted of the caregiver completing the identical assessment form and questionnaire that they had originally filled. As well, the subjects were given a second dental screening. 

The comparison group (CG; *n* = 181) was enrolled from the same centres used to enroll the study sample population but did not receive the anticipatory guidance presentation before data collection. The multiple choice questionnaire was completed at the beginning of the presentation session in order to determine the level of dental knowledge prior to any anticipatory guidance given by the researcher. After completion of the questionnaire, the dental screening for their children was completed. The presentation was provided at the end of the visit in order to provide anticipatory guidance without biasing the results of the data.

Summary statistics were computed using SAS version 9.2 for the study and comparison groups from the questionnaire and assessment form data. Chi-square tests and Fischer's exact tests were used to analyze data between the study group and the comparison group with regard to knowledge retention, presence of caries, and utilization of dental services.

## 3. Results

 The study group at baseline (SGB) consisted of 161 children. This cohort completed the study and was designated (SGFU; *n* = 161). The children's ages ranged from 0 to 31 months, the mean age being 17.6 months. Nine children included in the study group were not born at the time of the initial data collection. The study group followup (SGFU) consisted of 161 children. The mean age for the SGFU was 35.7 months, with an age range from 16 to 49 months. The comparison group (CG) was enrolled based on an age range that would be approximately comparable to the SGFU and consisted of 181 children. The mean age for the CG was 34.2 months, with an age range from 12 to 54 months.

As a measure of dental knowledge, a multiple choice questionnaire was administered to the caregivers of the study group at baseline (SGB), at followup (SGFU), and to the comparison group (CG). Using Chi-square and Fischer's exact test, each question on the multiple choice questionnaire was analyzed comparing the SGB with the CG as well as the SGB with the SGFU. The questionnaire responses revealed that in 20 of 23 questions, the SGB had a higher percentage of correct answers than the CG. The questions that showed a significant differences between the SGB and the CG pertained to the following topics: timing and frequency of oral hygiene practices, all questions related to fluoride, transmission of bacteria, breastfeeding, providing a safe home environment, and timing of the first dental visit.

Knowledge retention level of the study group at the follow-up period compared to their knowledge retention at baseline revealed a general trend for some loss of knowledge retention over the 18-month period. There was no significant loss of knowledge in the SGFU at a 5% significance level over the 18-month study period for 15 out of the 24 questions. The questions which showed a significant loss of knowledge over the 18-month time period were related to the following topics: timing of the first tooth, time required for toothbrushing, swallowing toothpaste, fluoridated water, transmission of bacteria, the role of breastfeeding in causing tooth decay, and timing of the first dental visit.

To determine the effectiveness of the anticipatory guidance model on preventable oral diseases, two methods of data collection were used. The first measure was the dental screening which included a record of visible caries, nonnutritive sucking habits, and trauma. Statistical analysis could only be performed for caries since the incidences were too low for other preventable oral conditions, such as trauma or nonnutritive sucking habits (NNSH). There was a significant difference on caries between the SGFU and the CG (*P* = 0.0001; Table 1). The control group at the end of the follow-up period had a caries prevalence of 6% as compared to 24% in the comparison group.

The second determinant for the effectiveness of this anticipatory guidance model on preventable oral disease was the assessment of high-risk behaviours. The results of the assessment form are shown in Table 2. Dental visits by caregivers: there was a higher percentage of caregivers in the SGFU (56.3%) that had themselves seen a dentist in comparison to the caregivers in the SGB (34.8%) and the CG (46.5%). Night time feeding practices: there was a dramatic decrease in the percentage of participants in the SGFU (16.1%) who allowed night time feeding for their children as compared to the SGB (40.4%) and the CG (47.8%).

The third objective of the study was to determine if anticipatory guidance had an effect on utilization of dental services. Chi-square analysis was performed on data gathered from the assessment form, in particular, the question related to having seen a dentist in the past. The follow-up answers of the study group compared to those of the comparison group are shown in Table 3. The results showed that there was a significantly higher degree of utilization of dental services by the study group participants as compared to those in the comparison group (*P* = 0.02). Of the 89% of the study group at baseline that had not utilized dental services, many responses were given as to the reason. The most frequent response given for the caregivers of the study group and the comparison group was that they were advised by a health care professional to go at a later age of their child (27.4% and 31.8%, resp.).

## 4. Discussion

### 4.1. Comprehensive Anticipatory Guidance Model

 An exhaustive review of the literature determined that there are no other audio-visual aids which discuss the full realm of anticipatory guidance topics for infant oral health. The unscripted, interactive aspect of the presentation was also beneficial to targeting the specific concerns of each group of caregivers and kept them engaged in the presentation. For example, presentations that included infants less than 12 months of age emphasized timing of tooth eruption. For groups with toddlers, proper home and car safety standards were emphasized. Previous studies have shown that tailored preventive education may have a longer impact than methods that are uniform [2].

### 4.2. Followup

 The study group baseline (SG) was contacted via telephone after an 18-month time period. Approximately 10% of caregivers that were contacted were not interested in continuing with the study because their child was already under the care of a dentist consequent to our initial presentation. While this was a negative aspect to data collection, it was a positive note for the ultimate goal of the study, that is, to increase dental utilization.

### 4.3. Utilization of Dental Services

 The results of this investigation showed that being advised by a health care professional was the most popular reason for not taking their child to a dentist. This highlights the issue that nondental health professionals need to be educated about the timing of the first dental visits for infants, so that the public receives a uniform message from all health professionals. Considering the limited number of paediatric dentists, it is important for the general dentist to provide access to this young patient population. General dentists should, at least, be comfortable screening children of this age group to assess their risk and determine their need for care by a paediatric dentist. Education programs have begun to address this issue at the undergraduate level and reinforce the importance of first dental visit at or before the child's first birthday.

### 4.4. Reduction in Early Childhood Caries

We saw a significant reduction in the incidence of caries in children whose parents were exposed to anticipatory guidance only once. The caries prevalence of 24% in the comparison group is approximately similar to those reported by public health, establishing this population as one at risk for dental disease. An even greater degree of reduction would have been noted with an intermediate recall at a 6–9 month time point. This observation is based on a much larger ongoing study which shows that children who are caries free are seen to be less likely to be brought back by their parents for routine follow-up visits at these free clinics. Reduction in the incidence of preventable oral disease is the ultimate goal of our model of anticipatory guidance and true test of its effectiveness. 

### 4.5. Questionnaire Responses

 The results showed that the SGB generally had more knowledge than the CG and that the SGFU generally had some loss of retention of that knowledge. It is interesting to note that the questions that showed a significant difference between the SGB and the CG were very similar to the questions that showed a significant loss of knowledge when comparing the SGB to the SGFU. Although it must be acknowledged that parents and caregivers who consented to volunteering in the study could be reasonably be assumed to be more motivated than those who did not, the loss of knowledge retention over the fairly long study period of 18 months demonstrates the attrition in recall of information and highlights areas of the presentation that need further clarity and reinforcement. Additionally, the loss in knowledge also demonstrated the reliability and validity of the questionnaire instrument used in this study and our previous study [8]. Some of these multiple choice questions showed no significant difference between the SGB and the CG and/or the SGFU, and some questions showed no loss of retention when comparing the SGB to the SGFU (*P* = 1.00) which suggests that caregivers may be receiving information about these topics from other sources.

### 4.6. Dental Screening

 The gold-standard for caries detection would have been a complete intraoral examination with mirror, explorer, overhead lighting, and radiographic examination if deemed necessary. However, Beltrán et al. [9] found in their evaluation of two methods for assessing oral health status that visual screenings gave data comparable to that produced from visual-tactile examinations. Screenings are used to seek out high-caries risk children and direct them to a dentist for further care; its purpose is not to replace a comprehensive oral examination.

### 4.7. Cost Effectiveness

 The use of existing community-oriented programs is a cost effective method of delivering anticipatory guidance to caregivers of infants and increasing access to dental care [10]. This model of delivering anticipatory guidance is more cost-effective than one-on-one counseling initiatives which are the most costly in terms of manpower, time, and financial resources, given the relatively few individuals that can be counseled. The model presented here may be a gateway program to allow parents to receive knowledge and learn whether their child is considered high risk and is in need for a dental visit [11] or for the modification of daily hygiene routines. The model of anticipatory guidance used in this study is cost effective as very few personnel and personnel hours are required to deliver the program. The widespread use of this model can be achieved and leads to an increase in access to care for certain under serviced populations.

### 4.8. Study Limitations and Recommendation for Future Use

 The language barrier was one limitation of the study. It is important to note that populations that cannot communicate fluently in English are likely the same populations that find it difficult to access care in the dental community. The audio-visual aid used in this study has been translated in to French, Spanish, and Arabic. It is recommended that the audio-visual aid be translated into many other languages and that multilingual personnel be trained to present this anticipatory guidance model. A second limitation to the study is access to dental services. It may have been helpful to give caregivers a list of private and public dental offices that are in their community.

## 5. Conclusions

1. There is some attrition in the retention of oral health knowledge over an 18-month time period suggesting that repeated reinforcement of the same principles and concepts might be advisable over a shorter time span.
2. A one-time exposure to anticipatory guidance has a positive effect on dental utilization. This underscores the importance of this model as a gateway into the dental system.
3. One time exposure to anticipatory guidance has an effect on caries incidence which underlines the importance of the timing of the model. This model, ideally, should be presented to the caregivers when the child is predentate.
4. This model of anticipatory guidance can provide long-term effectiveness in promoting the oral health of young children.

## Figures and Tables

**Table 1:** Chi-square analysis of caries in the SGFU and the CG.

	No caries	Caries	*P* value
SGFU	151	10	0.0001
CG	148	45	

There is a significant reduction in caries incidence among children whose families attended the “Baby Oral Health” model of anticipatory guidance.

**Table 2:** Summary of data collected from assessment form for the SGB, CG, and SGFU.

Questions asked	SGB		CG		SGFU	
	Yes (%)	No (%)	Yes (%)	No (%)	Yes (%)	No (%)
Pregnancy problems	13.2	86.8	11.1	88.9	12.9	87.1
Full term	78.4	21.6	90.7	9.3	90.3	9.7
Illness	14.6	85.4	18.2	81.8	12.5	87.5
Medications	9.7	90.3	4.3	95.7	6.3	93.8
Dental visits by caregiver	34.8	65.2	46.5	53.5	56.3	43.8
Breastfeeing	78.3	21.7	92.8	15.2	90.6	9.4
Nighttime feeding	40.4	59.6	47.8	52.2	16.1	83.9
Cup drinking	73.9	26.1	78.3	21.7	93.8	6.3
Special diet	10.0	90.0	7.0	93.0	0	100
Snacking	77.3	22.7	95.5	4.5	96.9	3.1
Fluoridated water usage	69.9	30.1	66.7	33.3	96.9	3.1
Bottled water usage	48.3	51.7	54.3	45.7	40.6	59.4
Fluoridated toothpaste	26.0	74.0	45.7	54.3	43.8	56.3
Pacifier	27.8	72.2	21.4	78.6	18.8	81.3
Digit habit	27.5	72.5	12.8	87.2	25.0	75.0
Walking	71.4	28.6	91.5	8.5	96.9	3.1
Injury	7.7	92.3	4.3	95.7	18.8	81.3
Teeth present	90.0	10.0	97.7	2.3	100	0
Teething problems	22.4	77.6	14.3	85.7	18.8	81.3
Clean mouth	78.9	21.1	91.5	8.5	90.6	9.4
Use of toothbrush	66.7	33.3	91.5	8.5	90.6	9.4
Use of toothpaste	57.5	42.5	90.7	9.3	83.9	16.1
Use of floss	6.3	93.8	21.4	78.6	15.6	84.4

**Table 3:** Chi-square analysis of dental utilization in the study group at followup and the comparison group.

	No dental visit	Dental visit	*P* value
Follow-up group	91	70	0.020
Comparison group	150	31	

There is a significant difference between utilization of dental services between the groups.
